# Comparative analysis of chloroplast genomes of *Pulsatilla* species reveals evolutionary and taxonomic status of newly discovered endangered species *Pulsatilla saxatilis*

**DOI:** 10.1186/s12870-024-04940-w

**Published:** 2024-04-17

**Authors:** Hefei Xue, Yanping Xing, Che Bian, Wenjuan Hou, Wenxiao Men, Han Zheng, Yanyun Yang, Xixiang Ying, Tingguo Kang, Liang Xu

**Affiliations:** 1 School of Pharmacy, Liaoning University of Traditional Chinese Medicine, Dalian, 116600 China; 2 Key Laboratory of Traditional Chinese Medicine Research and Development of Hebei Province, Institute of Traditional Chinese Medicine, Chengde Medical University, Chengde, 067000 China; 3 National Resource Center for Chinese Materia Medica, China Academy of Chinese Medical Sciences, Beijing, 100700 China; 4 State Key Laboratory of Dao-di Herbs, Beijing, 100700 China

**Keywords:** *Pulsatilla*, Plastome, Comparative analysis, Molecular markers, Phylogenetic tree

## Abstract

**Background:**

*Pulsatilla saxatilis*, a new species of the genus *Pulsatilla* has been discovered. The morphological information of this species has been well described, but its chloroplast genome characteristics and comparison with species of the same genus remain to be reported.

**Results:**

Our results showed that the total length of chloroplast (cp.) genome of *P. saxatilis* is 162,659 bp, with a GC content of 37.5%. The cp. genome contains 134 genes, including 90 known protein-coding genes, 36 tRNA genes, and 8 rRNA genes. *P. saxatilis* demonstrated similar characteristics to other species of genus *Pulsatilla*. Herein, we compared cp. genomes of 10 species, including *P. saxatilis*, and found that the cp. genomes of the genus *Pulsatilla* are extremely similar, with a length of 162,322–163,851 bp. Furthermore, The SSRs of *Pulsatilla* ranged from 10 to 22 bp in length. Among the four structural regions of the cp. genome, most long repeats and SSRs were detected in the LSC region, followed by that in the SSC region, and least in IRA/ IRB regions. The most common types of long repeats were forward and palindromic repeats, followed by reverse repeats, and only a few complementary repeats were found in 10 cp. genomes. We also analyzed nucleotide diversity and identified *ccs*A_*ndh*D, *rps*16_*trn*K-UUU, *ccs*A, and *rbc*L, which could be used as potential molecular markers for identification of *Pulsatilla* species. The results of the phylogenetic tree constructed by connecting the sequences of high variation regions were consistent with those of the cp. gene phylogenetic tree, and the species more closely related to *P. saxatilis* was identified as the *P. campanella*.

**Conclusion:**

It was determined that the closest species to *P. saxatilis* is *P. campanella*, which is the same as the conclusion based on pollen grain characteristics, but different from the *P. chinensis* determined based on morphological characteristics. By revealing information on the chloroplast characteristics, development, and evolution of the cp. genome and the potential molecular markers, this study provides effective molecular data regarding the evolution, genetic diversity, and species identification of the genus *Pulsatilla*.

**Supplementary Information:**

The online version contains supplementary material available at 10.1186/s12870-024-04940-w.

## Background

Pasque flower, the blooming of which symbolizes the early spring, is a distinctive alpine plant with very beautiful bell-shaped flowers. The most common pasque bears bluish-purple or dark violet flowers; however, its different cultivars offer a variety of color choices, bearing white and reddish-purple flowers [1]. According to plant taxonomy, pasque flower is classified as *Pulsatilla*. The genus *Pulsatilla* is primarily distributed in Europe and Asia. It is mostly used as a horticultural flower in Europe [2] and has a long history of medicinal use in China and other parts of Asia. As such, the roots of many *Pulsatilla* species, including *Pulsatilla chinensis* [3], *P. cernua, P. dahurica*, *and P. turczaninovii* [4], are listed in the pharmacopoeia or provincial medical standards for the treatment of dysentery and other conditions. The most distinctive feature of *Pulsatilla* is its long, feathery persistent style, appearing as an old man from afar, hence the name “Bai tou weng”.

**Fig. 1:**
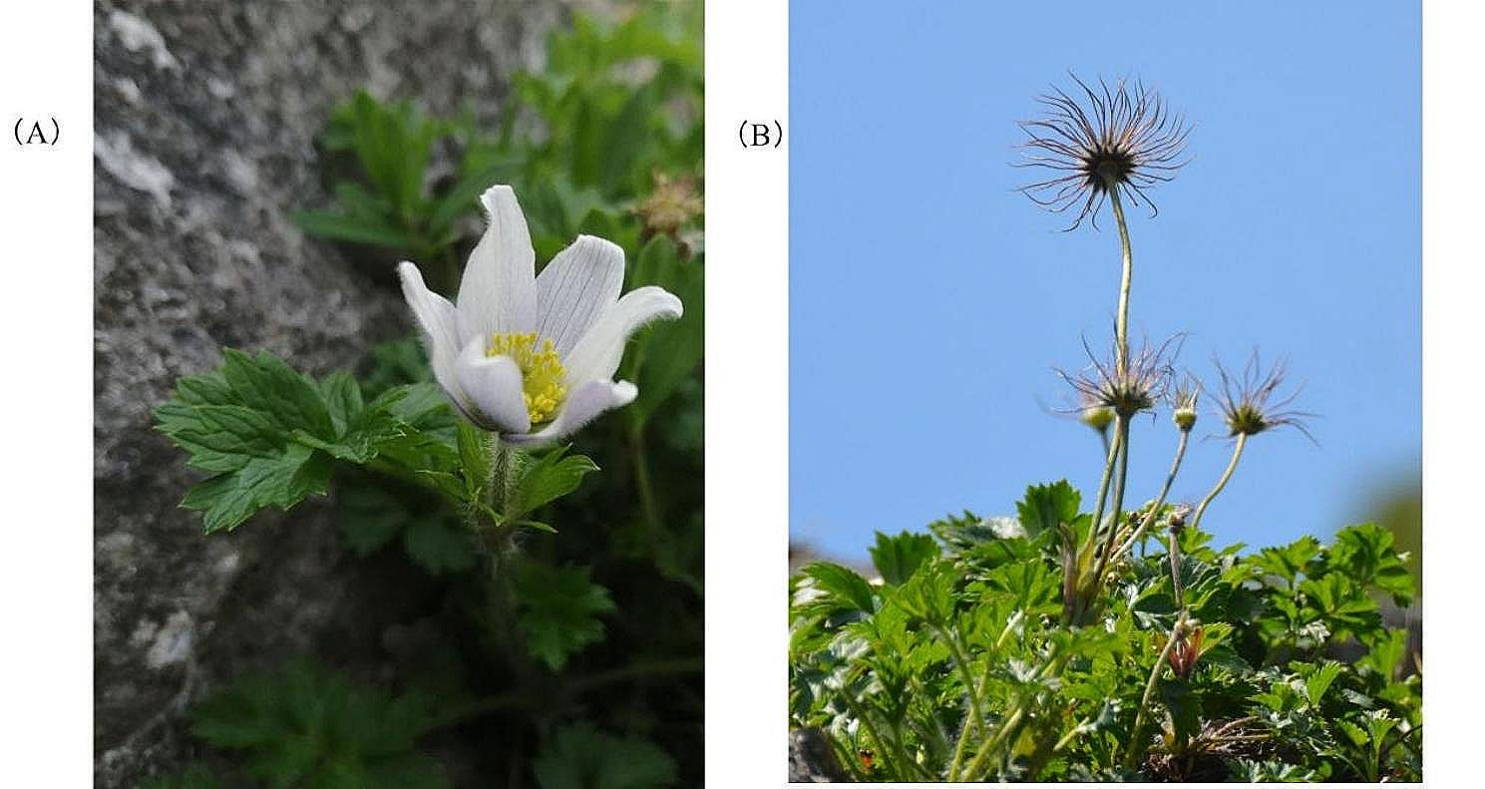
*Pulsatilla saxatilis* at flowering (**A**) and fruiting (**B**) stages

A new species of the genus *Pulsatilla, P. saxatilis* L.Xu & T.G.Kang (Fig. 1), was first discovered in the fourth Chinese materia medica resource inventory, where its primary morphological characteristics have been described in detail [5]. In morphology, *P. saxatilis* most closely resembles *P. chinensis*, with 3-foliolate leaves and solitary, erect flowers, only differing with respect to sepals of different colors and persistent styles of different lengths. The sepals of *P. saxatilis* are nearly white on the adaxial side and white to pale bluish-purple on the abaxial side, with the color of the base being the darkest, and the length of persistent style is 2–2.5 cm; these characteristics of *P. saxatilis* are distinct from those of *P. chinensis*, with purple sepals and persistent style being 3.5–6.5 cm long. The new species was discovered on a rocky cliff at an altitude of more than 1100 m on Baiyun Mountain in Fengcheng, Dandong, Liaoning Province. Owing to its highly narrow distribution area, it is being classified as endangered. Therefore, for the sake of ecological protection and basic research, it is crucial to take timely measures. This will not only promote the scientific development and utilization of *P. saxatilis* but will also prove significant for the species diversity and phylogenetic analysis of the genus *Pulsatilla*.

Plants have three sets of genomes: nuclear DNA, mitochondrial DNA (mtDNA), and chloroplast DNA (cpDNA). The nuclear genome contains abundant genetic information with significant genetic variation. The nuclear genome exhibits amphilepsis and bears a certain distinguishing ability for related species or subspecies. mtDNA is characterized by significant variation in genome size and structure, with the gene sequences being extremely conserved; they are the most conserved with the slowest evolution rate among those in the three sets of genomes. Owing to the lack of diversity, mtDNAs are typically not selected to be molecular markers for systematic studies. Furthermore, cpDNA generally comprises a covalently closed ring structure consisting of two large inverted repeats (IRs) as well as a large single-copy (LSC) and small single-copy (SSC) region [6]. The number and order of genes in cpDNA are relatively conservative, and its recombination is difficult to occur [7]. In most species, cpDNA is maternally inherited, has a relatively independent evolutionary path, and retains abundant genetic information in the evolutionary history [8]. Therefore, cpDNA has been widely used for studying plant genetic diversity, phylogeny, and evolution, as well as species identification and classification [9]. Phylogenetic trees constructed using only one or a combination of a few genes sometimes lack high resolution owing to insufficient loci information, horizontal gene transfer, presence of paralogous genes, and heterogeneity of gene evolution rate. Therefore, complete genome data is vital.

The primary reason of a species being endangered is the decrease in genetic diversity, which ultimately leads to a decrease in ecological adaptability. Therefore, an increasing number of studies are utilizing cp. genome to investigate the genetic diversity of endangered species and help establish protection measures [10]. Therefore, cp. genome can not only be used for species identification and molecular breeding research but also to provide a molecular basis for the improvement of yield and quality of important cash crops and horticultural varieties, as well as the protection of rare and endangered plants [11, 12].

Given that the genomic information and phylogeny of the newly discovered species *P. saxatilis* remain to be reported, herein, we sequenced and assembled cp. genome and annotated its genes and submitted the data to NCBI. This information is of vital significance to study the genetic and phylogenetic evolution, conservation, and development of *P. saxatilis*. The nine foreign species of *Pulsatilla* cp. genome [13], the information of which is submitted in NCBI, were excluded from this study owing to problems in gene assembly and failures in downloading and viewing the original data. Therefore, reliable, searchable genetic information from nine other species was included for comparison; eight of these species are mainly distributed in China and one in Korea. Moreover, the cp. genomes of *P. campanella* and *P. chinensis* f. *alba* were analyzed for the first time. By analyzing the cp. genome structure, codon usage preference, and simple sequence repeats (SSRs), sequencing differences, nucleotide diversity (Pi), and evolutionary selection pressure were compared, and phylogenetic trees were constructed. We further aimed to confirm that *P. saxatilis* is a new species and explored the phylogenetic position of *P. saxatilis* in genus *Pulsatilla*. Through phylogenetic and comparative analyses, we determined the relationship among the different species of the genus *Pulsatilla* in China and thus provided valuable information for the evaluation and determination of the medicinal varieties of the genus. The findings of this study will serve as a reference for the protection of endangered species and the exploitation and utilization of the medicinal resources of this genus.

## Results

### Basic characteristics of the cp. genome of *P. saxatilis*

**Fig. 2:**
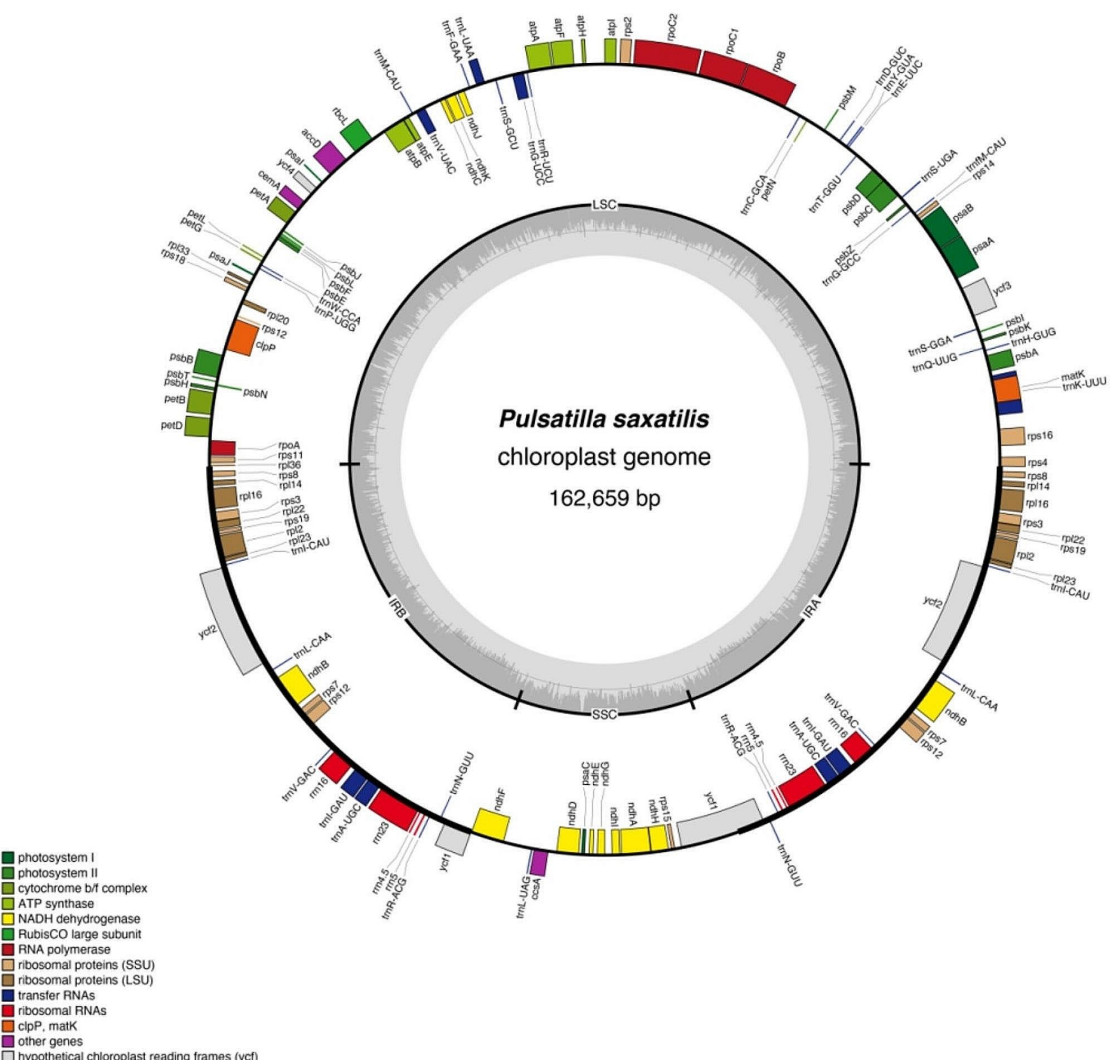
Annotation map of the cp. genome structure of *Pulsatilla saxatilis*. Genes placed outside the outer circle are transcribed clockwise, whereas those placed inside the outer circle are transcribed counter-clockwise. Genes belonging to different functional groups are color-coded. The gray histogram within the inner circle depicts the GC content of the genome, and the middle gray line indicates the 50% threshold line

In this study, we sequenced and reported the complete cp. genome of *P. saxatilis* for the first time. The entire genome was 162,659 bp in length and had a typical circular structure, with a GC content of 37.5%. Additionally, the complete cp. genome of *P. saxatilis* (shown in Fig. 2) consisted of two inverted repeat regions (IRA and IRB), an LSC region (82,225 bp), and an SSC region (17,848 bp). The genome contained 134 genes, including 90 known protein-coding genes (PCGs), 36 tRNA genes, and 8 rRNA genes. The total length of the coding gene was 94,918 bp, accounting for 58.35% of the total length of the genome. Genes annotated in the *P. saxatilis* cp. genomes are listed in Table 1. Moreover, the *rps*12 gene was trans-spliced.

**Table 1:** Genes annotated in the cp. genomes of *Pulsatilla saxatilis*

Category	Function	Gene name
Photosynthesis	Subunits of ATP synthase (6)	*atpA*, *atpB*, *atpE*, atpF*, *atpH*, *atpI*
	Subunits of NADH dehydrogenase (12)	*ndhA* *, *ndhB* *^#^, *ndhC*, *ndhD*, *ndhE*, *ndhF*, *ndhG*, *ndhH*, *ndhI*, *ndhJ*, *ndhK*
	Subunits of cytochrome (6)	*petA*, *petB* *, petD *, *petG*, *petL*, *petN*
	Subunits of photosystem I (5)	*psaA*, *psaB*, *psaC*, *psaI*, *psaJ*
	Subunits of photosystem II (15)	*psbA*, *psbB*, *psbC*, *psbD*, *psbE*, *psbF*, *psbH*, *psbI*, *psbJ*, *psbK*, *psbL*, *psbM*, *psbN*, *psbT*, *psbZ*
Transcription and translation	Large subunit of ribosome (13)	*rpl2* *^#^, *rpl14*^#^, *rpl16* *^#^, *rpl20*, *rpl22*^#^, *rpl23*^#^, *rpl33*, *rpl36*
	DNA dependent RNA polymerase (4)	*rpoA*, *rpoB*, *rpoC1* *, *rpoC2*
	Small subunit of ribosome (17)	*rps2*, *rps3*^#^, *rps4*, *rps7*^#^, *rps8*^#^, *rps11*, *rps12* *^#1^, *rps14*, *rps15*, *rps16* *, *rps18*, *rps19*^#^
	rRNA Genes (8)	*rrn4.5*^#^, *rrn5*^#^, *rrn16*^#^, *rrn23*^#^
	tRNA Genes (36)	*trnA*-*UGC* *^#^, *trnC*-*GCA*, *trnD*-*GUC*, *trnE*-*UUC*, *trnF*-*GAA*, *trnG*-*GCC*, *trnG*-*UCC* *, *trnH*-*GUG*, *trnI*-*CAU* *^#^, *trnI*-*GAU*^#^, *trnK*-*UUU* *, *trnL*-*CAA*^#^, *trnL*-*UAA* *, *trnL*-*UAG*, *trnfM*-*CAU*, *trnM*-*CAU*, *trnN*-*GUU*^#^, *trnP*-*UGG*, *trnQ*-*UUG*, *trnR*-*ACG*^#^, *trnR*-*UCU*, *trnS*-*GCU*, *trnS GGA*, *trnS*-*UGA*, *trnT*-*GGU*, *trnV*-*GAC*^#^, *trnV*-*UAC* *, *trnW*-*CCA*, *trnY*-*GUA*
Other genes	Subunit of rubisco (1)	*rbcL*
	Subunit of Acetyl-CoA-carboxylase (1)	*accD*
	c-type cytochrome synthesis gene (1)	*ccsA*
	Envelop membrane protein (1)	*cemA*
	Protease (1)	*clpP* **
	Maturase (1)	*matK*
Unknown function	Conserved open reading frames (6)	*ycf 1*^#^, *ycf 2*^#^, *ycf 3* **, *ycf 4*

*Note* *genes containing one intron, **genes containing two introns, #gene in the IR region with two copies present, ^1^ trans-spliced genes

### Comparative analysis of the cp. genome of *Pulsatilla*

The complete cp. genomes of 10 *Pulsatilla* species ranged from 162,322 bp (*P. campanella*) to 163,851 bp (*P. chinensis*) in length, with a maximum difference of 1,529 bp and a minimum difference of 31 bp. The size of the LSC region ranged from 81,894 bp (*P. dahurica*) to 82,606 bp (*P. tongkangensis*), with a maximum difference of 712 bp and a minimum difference of 29 bp. Moreover, the size of the SSC region ranged from 17,497 bp (*P. campanella*) to 19,272 bp (*P. chinensis*), with a maximum difference of 1,775 bp and a minimum difference of 5 bp. The size of the IR region varied from 31,084 bp (*P. tongkangensis*) to 31,410 bp (*P. cernua*), with a maximum difference of 326 bp and a minimum difference of 1 bp. Furthermore, the GC content ranged from 37.1 to 37.5%. Additionally, we found that the number of genes remained consistent in all the species. A total of 134 genes were observed, including 36 tRNAs, 8 rRNAs, and 90 PCGs, wherein 14 tRNAs and 8 rRNAs were located in the IR region. On comparing the cp. gene characteristics in seven species and three subspecies units of *Pulsatilla*, we found that the cp. genes were highly conserved (Table 2), and the relative positions and sizes of different genes were similar in the 10 species.

**Table 2:** Summary of cp. genome characteristics of 10 *Pulsatilla* species

Species	Size (bp)	GenBank Accession No.	GC Content (%)	LSC length (bp)	SSC length (bp)	IR length (bp)	Gene number	Protein-coding gene number	rRNA gene number	tRNA gene number
*P. saxatilis*	162,659	OP729488	37.5	82,225	17,848	31,293	134	90	8	36
*P. chinensis*	163,851	MK860682	37.1	82,342	19,272	31,118	134	90	8	36
*P. cernua*	162,924	MK860687	37.5	82,427	17,676	31,410	134	90	8	36
*P. dahurica*	162,450	MK860685	37.4	81,894	17,843	31,356	134	90	8	36
*P. turczaninovii*	162,795	MK860686	37.4	82,177	18,243	31,187	134	90	8	36
*P. campanella*	162,322	OL450399	37.4	82,087	17,497	31,369	134	90	8	36
*P. tongkangensis*	163,442	OM328110	37.3	82,606	18,668	31,084	134	90	8	36
*P. chinensis* var. *kissii*	163,756	MK860683	37.2	82,294	19,224	31,119	134	90	8	36
*P. cernua* f. *plumbea*	162,481	MK860684	37.4	81,923	17,871	31,343	134	90	8	36
*P. chinensis* f. *alba*	163,539	ON920514	37.2	82,255	19,054	31,115	134	90	8	36

The overall sequence identity of the cp. genomes of the 10 *Pulsatilla* species was plotted using mVISTA, with the annotation of *P. chinensis* as a reference. The 10 species demonstrated high sequence similarity, and the IR region was more conserved than the single-copy region. Genetic variation in the intergenic spacers (IGS) was more common than in the coding regions. The coding region has three regions of high difference, namely *ycf*1 (refers to *ycf*1 located in the SSC-IRA region), *ycf*2, and *ndh*F. Moreover, significant differences were observed in the IGS, and the highly variable IGS regions included *rps*16_*trn*K-UUU, *trn*Y-GUA_*trn*D-GUC, *ndh*C_*trn*V-UAC, *ndh*F_*trn*L-UAG, and *ccs*A_*ndh*D.

**Fig. 3:**
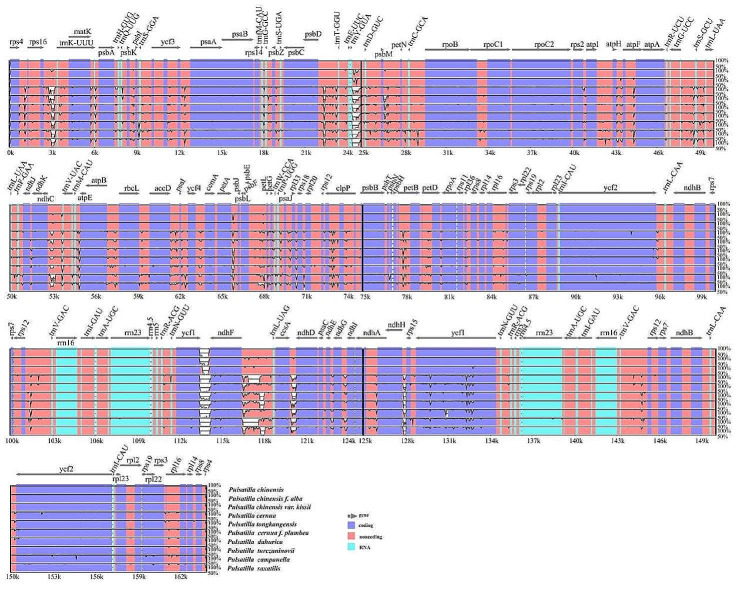
Visualization alignment of the cp. genome sequence of 10 *Pulsatilla* species, using *P. chinensis* as the reference sequence. The identity percentages ranging from 50–100% are shown on the y-axis, whereas the positions within the cp. genome are shown on the x-axis. Each arrow indicates the annotated genes and direction of their transcription in the reference genome. Genome regions, i.e., coding sequences, noncoding sequences, and RNA, are color-coded

To further understand the differences among the cp. genome sequences of 10 *Pulsatilla* species, we used the nucleotide substitution number and sequence Kimura 2-parameter (K2p) distance to indicate the degree of differences. The nucleotide substitution number of 10 species ranged from 17 to 1308, and the K2p distance ranged from 0.0001 to 0.00816 (Table 3). *P. chinensis* var. *kissii* and *P. chinensis* demonstrated the smallest sequence difference, with *P. dahurica* and *P. campanella* exhibiting the largest sequence difference.

**Table 3:** Number of nucleotide substitutions and sequence distance in 10 complete cp. genomes

	*P.chinensis*	*P. chinensis f. alba*	*P. chinensis var. kissii*	*P. cernua*	*P. tongkangensis*	*P. cernua f. plumbea*	*P.dahurica*	*P. turczaninovii*	*P. campanella*	*P. saxatilis*
*P. chinensis*		0.00032	0.0001	0.00477	0.004	0.00417	0.00414	0.00441	0.00742	0.00657
*P. chinensis f. alba*	52		0.00025	0.00465	0.00399	0.0042	0.00422	0.00446	0.00745	0.00659
*P. chinensis var. kissii*	17	41		0.00426	0.00391	0.00426	0.00423	0.00447	0.00748	0.00658
*P. cernua*	769	749	689		0.00282	0.00402	0.00388	0.00382	0.00784	0.00703
*P. tongkangensis*	648	646	633	456		0.00282	0.00293	0.00419	0.00743	0.0062
*P. cernua f. plumbea*	673	678	689	648	456		0.00017	0.00382	0.00813	0.00615
*P. dahurica*	669	681	683	625	473	28		0.00384	0.00816	0.00623
*P. turczaninovii*	714	721	724	616	679	616	619		0.00705	0.00576
*P. campanella*	1190	1195	1199	1259	1192	1302	1308	1129		0.00516
*P. saxatilis*	1057	1061	1059	1129	999	989	1003	927	830	

The upper triangle shows the number of sequence distance in complete cp. genomes and the lower triangle indicates the number of nucleotide substitutions

### Nucleotide diversity (pi)

**Fig. 4:**
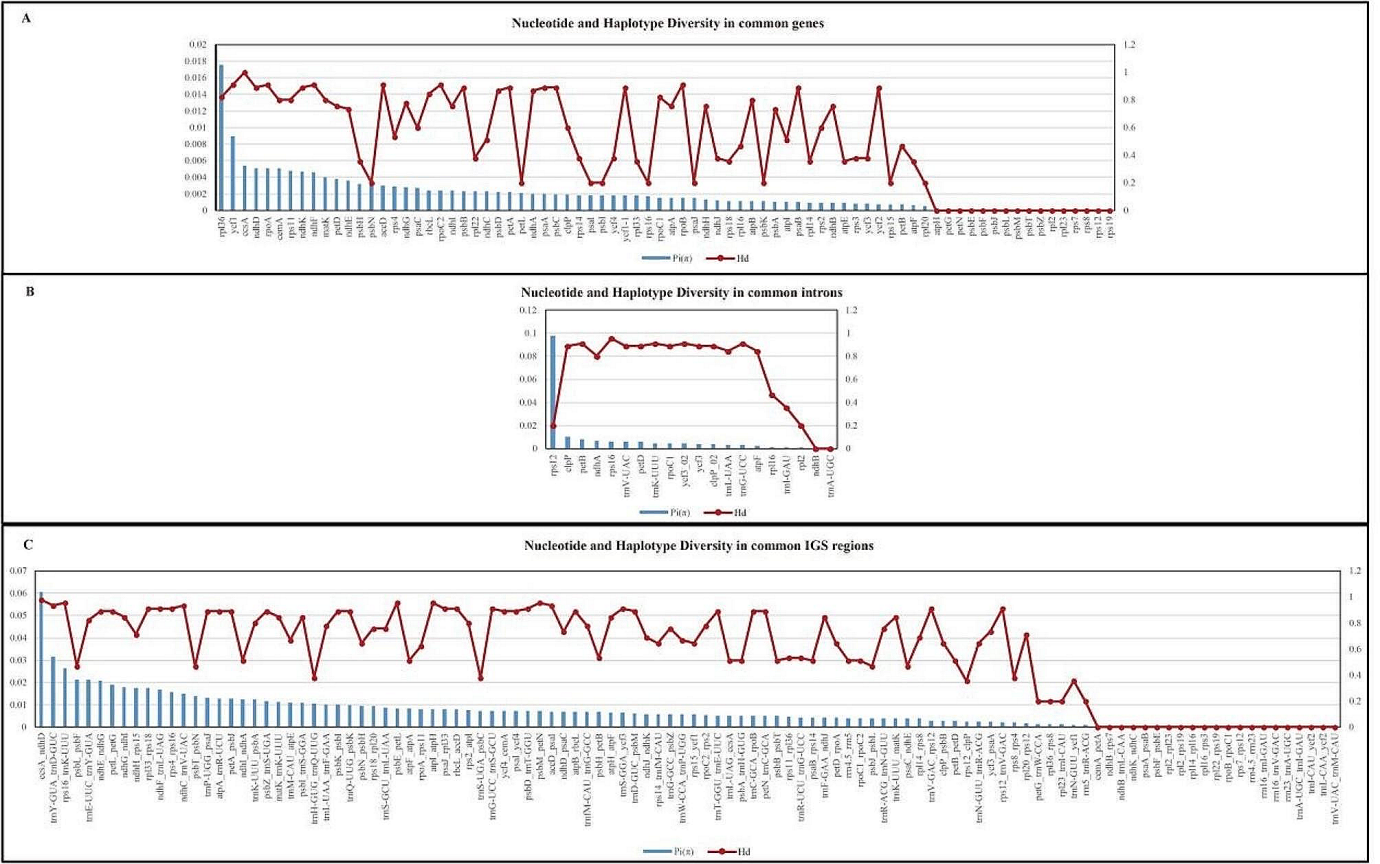
Nucleotide and haplotype diversity of cp. genomes of *Pulsatilla*

Nucleotide and haplotype diversity and GC content of 78 genes, gene introns, and IGS regions in the cp. genome of 10 *Pulsatilla* species were calculated (Fig. 4, Table S1). Among the 78 genes, *rpl*36 demonstrated the highest Pi value of 0.01754. The genes with high Pi values were primarily distributed in the LSC region and those with moderate and low Pi values in the SSC and IRA or IRB regions, respectively, indicating that the IR region is extremely conserved and less sensitive to the evolutionary pressure of these genes. In the gene intron region, the Pi value of *rps*12 intron was up to 0.09763 and that of other introns was similar to that in the gene region. Moreover, we observed high nucleotide diversity in the IGS region, with *ccs*A_*ndh*D exhibiting a maximum Pi of 0.06066 and IGS regions demonstrating much higher Pi values than gene regions. The intron, IGS region, and coding region genes with high Pi values and three universal cp. DNA barcodes *psb*A_*trn*H, *mat*K, and *rbc*L are listed in Table 4.

**Table 4:** Variability of seven variable markers and universal cp. DNA barcodes (*rbc*L, *mat*K, and *psb*A_*trn*H) in *Pulsatilla*

Gene ID	G + C tot	Pi(π)	Hap	Hd	Position	Type
*rps12*	0.3815	0.09763	2	0.2	IRA/IRB	introns
*ccsA_ndhD*	0.1376	0.06066	9	0.978	SSC	IGS regions
*trnY_trnD*	0.2387	0.03161	7	0.933	LSC	IGS regions
*rps16_trnK*	0.2789	0.02646	8	0.956	LSC	IGS regions
*rpl36*	0.4123	0.01754	4	0.822	LSC	CDS
*ycf1*	0.3118	0.00896	7	0.911	SSC-IRA	CDS
*ccsA*	0.316	0.00542	10	1	SSC	CDS
*psbA_trnH*	0.3244	0.00518	3	0.511	LSC	IGS regions
*matK*	0.3147	0.00404	5	0.8	LSC	CDS
*rbcL*	0.4439	0.00244	5	0.844	LSC	CDS

### Codon usage bias

Codon bias refers to the frequency of codon usage in protein translation, which is affected by several factors such as gene mutation and nucleotide composition. Codon usage is typically assessed using relative synonymous codon usage (RSCU), the number of codon usage, and the fraction of codon for each amino acid.

The cpDNA of *P. saxatilis* contains 27,709 codons, encoding a total of 20 amino acids. From codon classification analysis, we found that the most commonly encoded amino acid was leucine (Leu), with a total of 2831 codons (10.22%), including 6 synonymous codons, of which the UUA codon was the most common (Table 5, supplementary Fig. S1). It was followed by isoleucine (Ile, 8.69%), serine (Ser, 7.59%), glycine (Gly, 6.76%), arginine (Arg, 6.13%), and phenylalanine (Phe, 5.50%). Cystine (Cys, 328) was the least encoded amino acid. Of these codons, AUU (codon 1162) encoded isoleucine (Ile) and UGC (codon 95) encoded cysteine were the most and least frequently used codons (Table 5).

The codon usage in cp. genomes of the 10 species was highly similar. All identified 30 codons exhibited an RSCU value greater than 1, indicating a preference for their codon usage (Table 5). In the third position, 16 codons ended in U(T), 13 ended in A, and only one ended in G, indicating the strong A/T preference of the codons of *Pulsatilla* cpDNA in the third position. Additionally, the RSCU values of the start codon AUG and trp coding codon UGG were both 1, indicating no preference, whereas the RSCU value of the stop codon UAA was greater than 1, indicating a preference.

**Table 5:** Relative synonymous codon usage (RSCU) for protein-coding genes in *Pulsatilla saxatilis*

Codon	AA	ObsFreq	RSCU	Codon	AA	ObsFreq	RSCU	Codon	AA	ObsFreq	RSCU
GCA	Ala	419	1.13	AAG	Lys	391	0.53	AGC	Ser	131	0.37
GCC	Ala	251	0.68	CUA	Leu	387	0.82	AGU	Ser	392	1.12
GCG	Ala	189	0.51	CUC	Leu	206	0.44	UCA	Ser	447	1.27
GCU	Ala	618	1.67	CUG	Leu	188	0.4	UCC	Ser	348	0.99
UGC	Cys	95	0.59	CUU	Leu	602	1.27	UCG	Ser	212	0.6
UGU	Cys	229	1.41	UUA	Leu	853	1.81	UCU	Ser	579	1.65
GAC	Asp	233	0.4	UUG	Leu	598	1.27	ACA	Thr	441	1.24
GAU	Asp	925	1.6	AUG	Met	658	1	ACC	Thr	253	0.71
GAA	Glu	1068	1.47	AAC	Asn	309	0.46	ACG	Thr	171	0.48
GAG	Glu	385	0.53	AAU	Asn	1045	1.54	ACU	Thr	558	1.57
UUC	Phe	554	0.72	CCA	Pro	336	1.15	GUA	Val	562	1.51
UUU	Phe	984	1.28	CCC	Pro	230	0.78	GUC	Val	190	0.51
GGA	Gly	742	1.58	CCG	Pro	159	0.54	GUG	Val	206	0.55
GGC	Gly	199	0.42	CCU	Pro	448	1.53	GUU	Val	535	1.43
GGG	Gly	327	0.7	CAA	Gln	736	1.51	UGG	Trp	481	1
GGU	Gly	608	1.3	CAG	Gln	239	0.49	UAC	Tyr	202	0.4
CAC	His	157	0.46	AGA	Arg	507	1.79	UAU	Tyr	817	1.6
CAU	His	526	1.54	AGG	Arg	193	0.68	UAA	*	44	1.47
AUA	Ile	762	0.95	CGA	Arg	394	1.39	UAG	*	25	0.83
AUC	Ile	486	0.61	CGC	Arg	112	0.39	UGA	*	21	0.7
AUU	Ile	1159	1.44	CGG	Arg	130	0.46				
AAA	Lys	1089	1.47	CGU	Arg	368	1.3				

### Analysis of evolutionary selection pressure

**Fig. 5:**
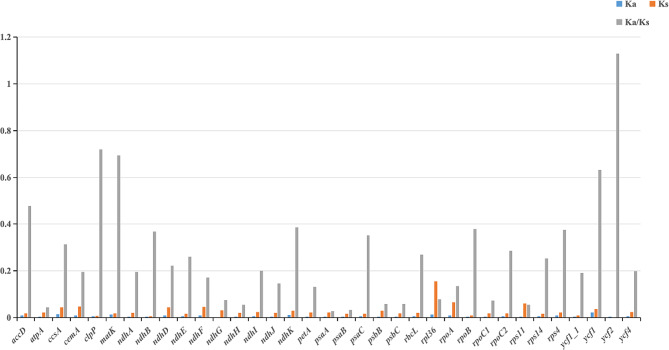
Ka, Ks values of 35 protein-coding genes

Nonsynonymous substitution rate (Ka), synonymous substitution rate (Ks) and Ka/Ks ratio are commonly used to evaluate the evolution rate between gene sequences and better elucidate whether selection pressure is associated with a particular protein-coding gene. The Ka/Ks values of 78 protein-coding genes in the cp. genome of 10 *Pulsatilla* genus were calculated. The results showed that Ka of most genes was less than Ks value, that is, Ka/Ks < 1, which means that most protein-coding genes have been purified in the process of evolution. Only *ycf*2 gene showed Ka/Ks > 1, and the gene showed positive selection effects. (Fig. 5, supplementary Table S2).

### Analysis of long repeats and SSRs

**Fig. 6:**
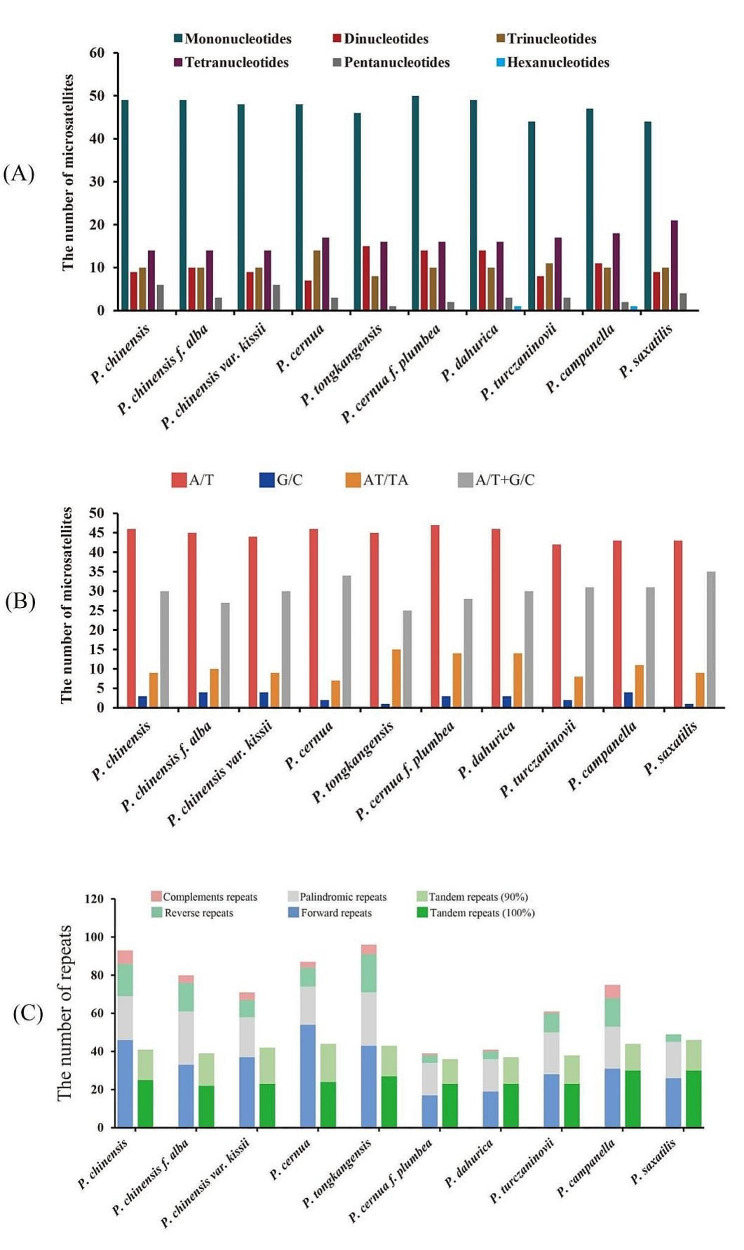
Repeat sequence analysis of *Pulsatilla*. (**A**) Frequency of SSRs by types; (**B**) frequency of SSRs by base composition; (**C**) number of five repeat types

Our findings showed that the most common types of long repeats were forward and palindromic repeats, followed by reverse repeats, and only a few complementary repeats were found in 10 cp. genomes. Most repeats were short, ranging from 30 to 49 bp in length. Most mononucleotide consisted of A/T, and other SSR types mainly included AT/TA, with little G/C. Each of the 10 cp. genomes consisted of 83–93 SSRs. These SSR ranged from 10 to 22 bp in length. Among the four structural regions of the cp. genome, most long repeats and SSRs were detected in the LSC region, followed by that in the SSC region, and least in IRA/ IRB regions. The above results are shown in Fig. 6.

### Phylogenetic analysis

**Fig. 7:**
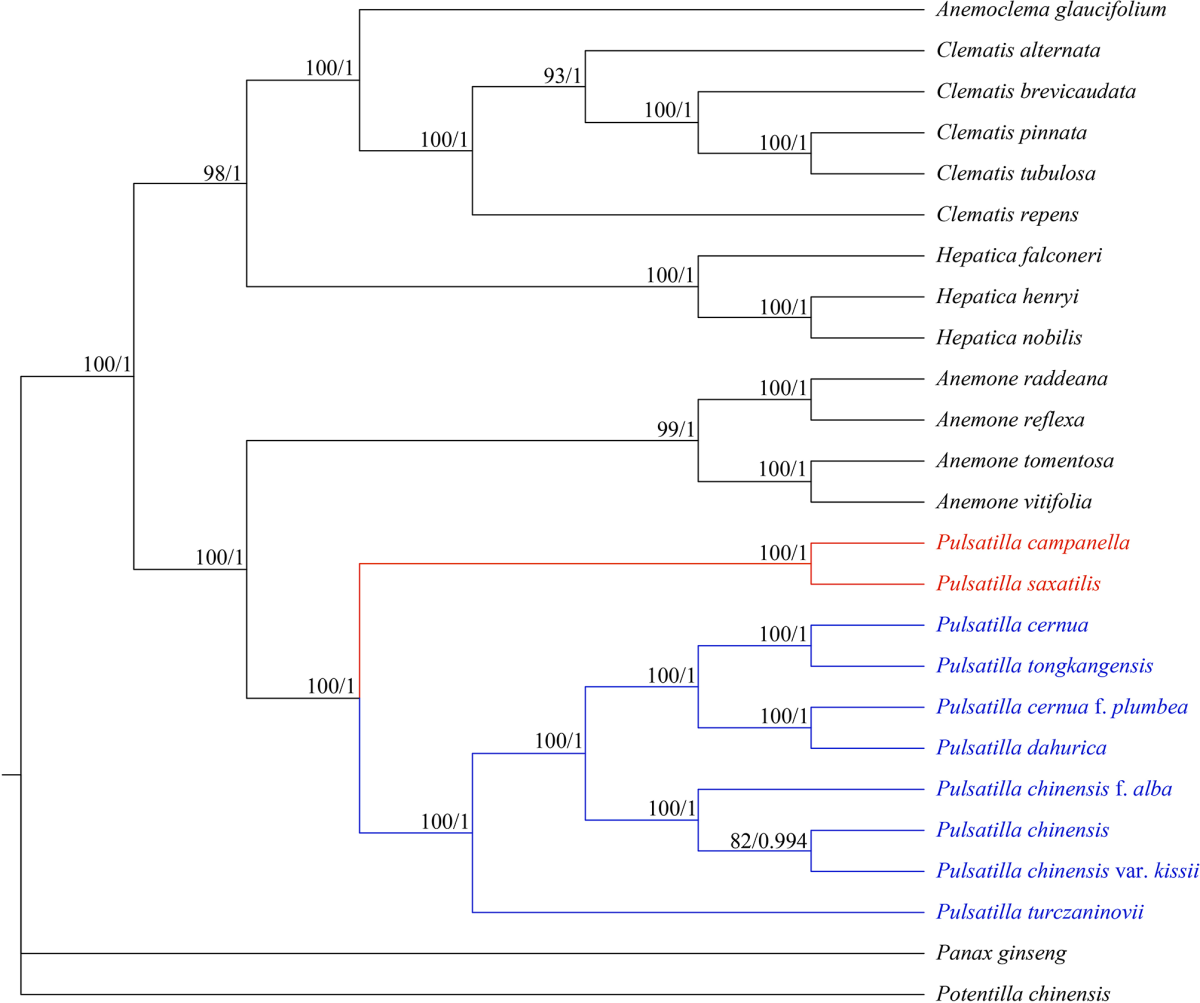
Phylogenetic tree of *Pulsatilla saxatilis* and 24 other species using maximum likelihood (ML) and Bayesian inference methods based on the complete cp. genome sequences. Number of the branches indicates ML bootstrap support value/Bayesian posterior probability

Phylogenetic trees were constructed for 10 *Pulsatilla* species and 13 other Ranunculaceae species using the complete cp. genome (Fig. 7). The best amino acid substitution model was selected using GTR + F + I + G4, with *Potentilla chinensis* of the *Rosaceae* family and *Panax ginseng* of the *Araliaceae* family as outgroup clusters. The phylogenetic tree generated 22 nodes, with most of them exhibiting perfect bootstrap and Bayesian test post-probability support. The result of maximum likelihood (ML) phylogenetic tree is presented in Fig. 7. The topology of Bayesian inference (BI) tree was consistent with that of ML tree. *P. saxatilis* formed a sister relationship with *P. campanella* (red), whereas the most morphologically similar *P. chinensis* was resolved on a more distant phylogenetic position. Coupled with morphological characteristics, the phylogenetic results further verified that *P. saxatilis* is a separate, new species.

**Fig. 8:**
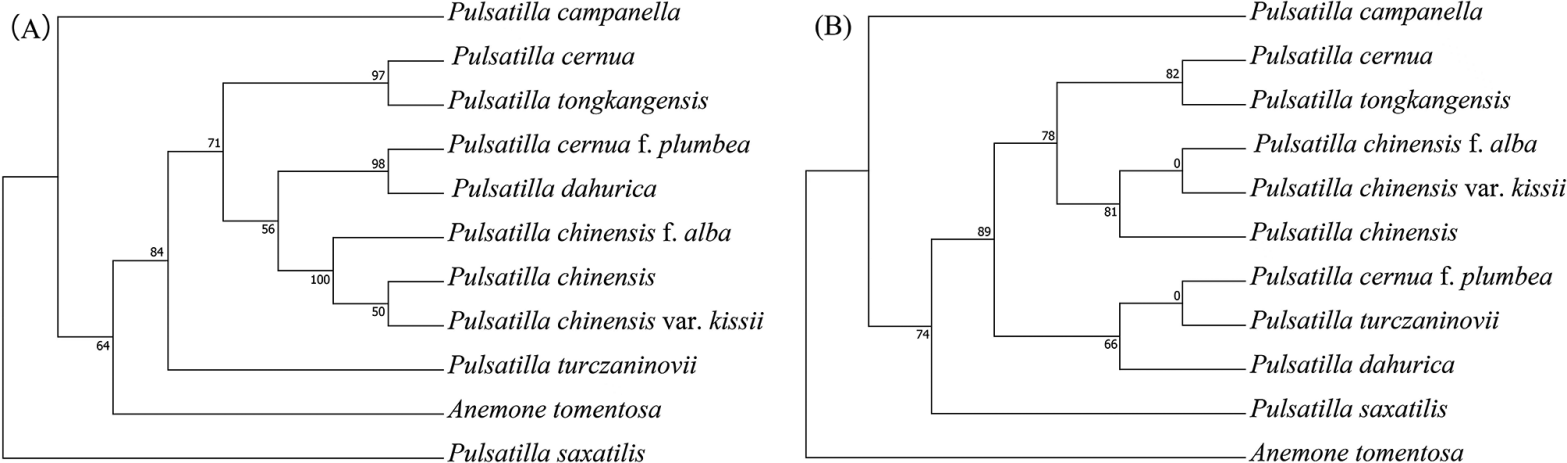
Phylogenetic trees of 10 *Pulsatilla* species and outgroup *Anemone tomentosa* using maximum likelihood (ML) based on *rps*16_*trn*K-UUU (**A**) and *rbc*L (**B**). Number of the branches indicates ML bootstrap support value

**Fig. 9:**
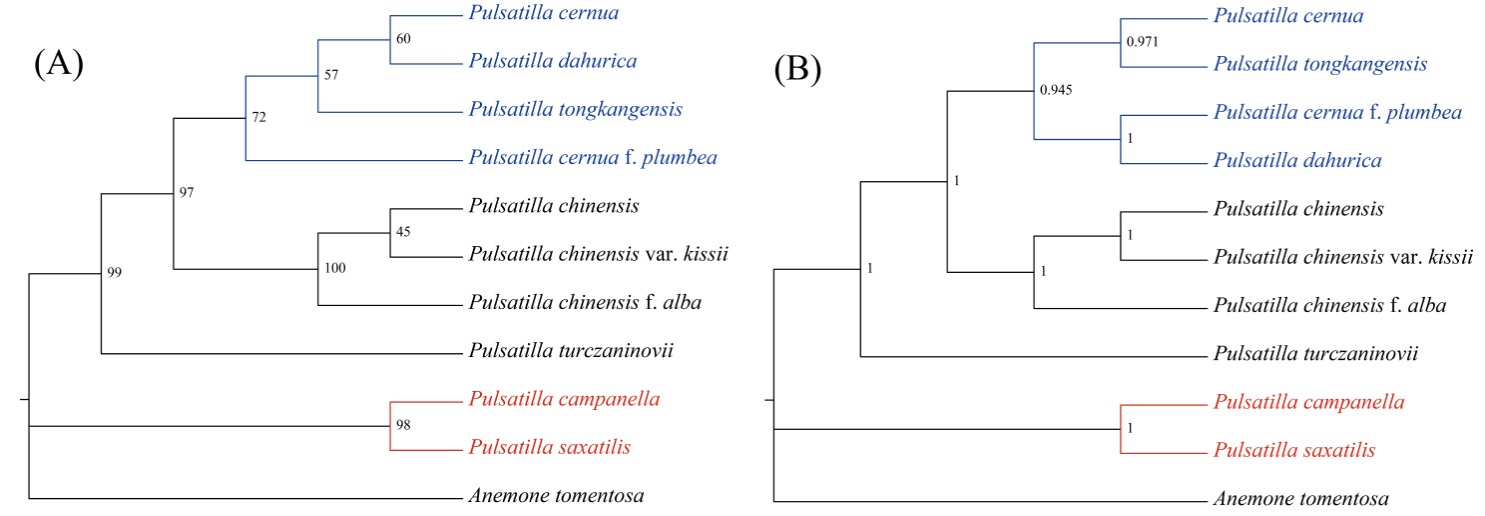
Phylogenetic tree of 10 *Pulsatilla* species and outgroup *Anemone tomentosa* using maximum likelihood (ML) (**A**) and Bayesian inference methods (**B**) based on the high variable region sequences. Number of the branches indicates ML bootstrap support value/Bayesian posterior probability. Red branches are for *P. saxatilis* and its sister taxa, and blue marks the different branches between the two trees

Ten sequences in Table 4 were extracted from cp. gene respectively, used MAFFT v7 to align and the best amino acid substitution model of ML tree were selected to build phylogenetic trees. The phylogenetic trees of *rps*16_*trn*K-UUU (A) and *rbc*L (B) were shown in Fig. 8, phylogenetic trees built based the other eight sequences were shown in Figure S3. At the same time, according to the analysis results of nucleotide and haplotype diversity, the following six sequences exhibited the highest Pi values: *rps*12-intron, *ccs*A_*ndh*D, *psb*L_*psb*F, *rps*16_*trn*K-UUU, *trn*E-UUC_*trn*Y-GUA, and *trn*Y-GUA_*trn*D-GUC. We used MAFFT v7 to align the six sequences separately and subsequently concatenate the sequences. Fuzzy areas were trimmed using Gblocks contrast, and the resulting sequences were used to build phylogenetic trees. The best amino acid substitution model of BI tree was GTR + F + G4. Our finding showed that the BI tree demonstrated the same topological structure as the phylogenetic tree based on the cp. genome, and all branches had high support values (Fig. 9B). The best amino acid substitution model of ML tree was F81 + F. Moreover, the ML tree differed (Fig. 9A) from the first two trees, the branch formed of *P. cernua*, *P. tongkangensis*, *P. cernua* f. *plumbea*, and *P. dahurica* had a different relationship, and the bootstrap support was slightly lower.

### Time estimation

**Fig. 10:**
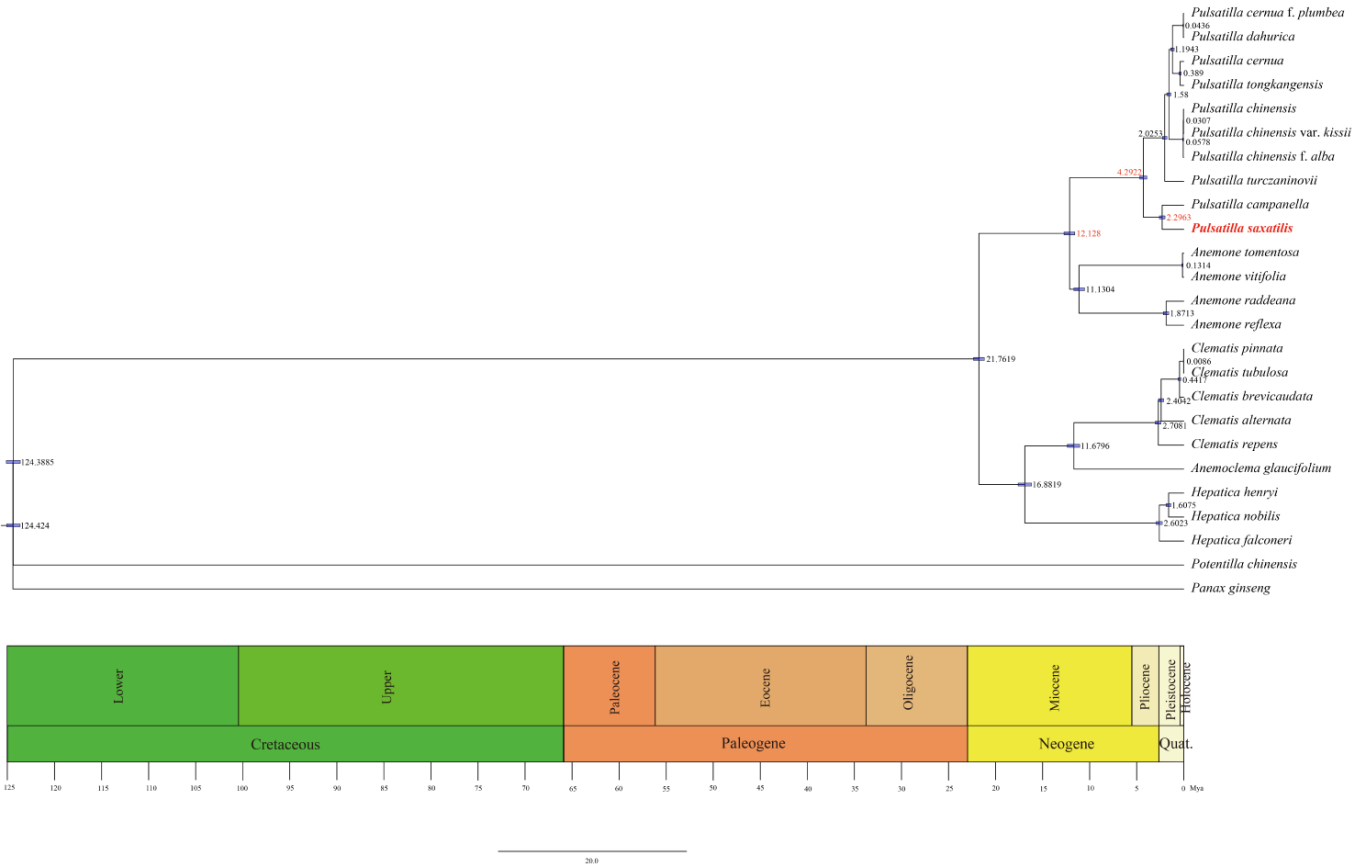
Divergence times of 10 *Pulsatilla* species obtained from BEAST analysis based on the complete cp. genome sequences. Mean divergence time of the nodes were shown next to the nodes while the blue bars correspond to the 95% highest posterior density (HPD)

After ML phylogenetic reconstruction, a divergence time was established, with *Panax ginseng* (KM067390) as a root species, estimated to be 124 Mya. The monophyletic group to which the genus *Pulsatilla* belongs diverged at about 21.7619 Mya, *Pulsatilla* diverged from *Anemone* at about 12.128 Mya. The isolation of the clade of species within the genus *Pulsatilla* occurred at about 4.2922 Mya. The division between *Pulsatilla saxatilis* and *Pulsatilla campanella* occurs around 2.2963 Mya (Fig. 10).

## Discussion

### Comparative analysis of the cp. genome of *Pulsatilla*

From the visualization alignment of the cp. genome sequence of *Pulsatilla* (Fig. 3), it can be intuitively seen that the cp. genomes of different *Pulsatilla* species had similar structure and gene sequence. Moreover, the divergence level in the noncoding region was higher than that in the coding region. Additionally, the difference between the LSC and SSC regions was greater than that between the IR regions. Sequence divergence in the single copy region was higher than that in the IR region and that in the noncoding region was higher than that in the coding region. These results were similar to those previously reported in the genus *Pulsatilla* [14, 15].

### Gene codon usage in the cp. genome of *Pulsatilla*

Single nucleotide polymorphisms (SNPs) appear most frequently in CG sequences, and C to T polymorphisms are the most common transitions, because C in CG is often methylated and becomes thymine after spontaneous deamination. Generally, SNPs are single nucleotide variants with a frequency greater than 1%. According to the criteria proposed by Grant et al. (1998), the critical value of haplotype diversity was 0.5 and that of nucleotide diversity was 0.005. The higher the value of both, the higher is the degree of population diversity.

Genes in the chloroplasts are functionally important and might have been under selection during evolution. To analyze the selection pressure on the cp. genome of *Pulsatilla*, we calculated the non-synonymous/synonymous replacement rates (Ka/Ks) of the coding genes of 10 *Pulsatilla* species. Genes with Ka/Ks values higher than 1.0 should be selected positively and may be candidates for functional adaptation, while genes below 1.0 should be selected negatively [16]. Most genes had Ka/Ks values below 1.0 (Fig. 5), reflecting selective pressure to maintain gene function. According to the calculation, only *ycf*2 Ka/Ks > 1, and *clp*P, *mat*K and *ycf*1 were 0.5-1. The rest are 0-0.5. It has been reported that genes involved in photosystems(*psb*D, *psb*E, *psb*F, *psb*I, *psb*J, *psb*K, *psb*L, *psb*M, *psb*N, *psb*T, *psb*Z)the cytochrome b/f complex (*pet*B, *pet*D, *pet*G, *pet*L, *pet*N), and some ATP synthases (*atp*B, *atp*E, *atp*F, *atp*H, *atp*I) in all species have Ka/Ks values close to 0 [17]. The genes with high ratio were unclassified genes (*ycf*1, *ycf*2), and enzyme related genes (*clp*P, *mat*K). The cp. genes of *Pulsatilla* have high conservation.

### Phylogenetic analysis of the Genus *Pulsatilla*

Phylogenetic trees were constructed for 10 species of genus *Pulsatilla*, and they were color-coded to reflect clade identity. Among them, *P. chinensis* and its variety *P. chinensis* var. *kissii* were first clustered into a branch, which was then clustered into a branch with its forma *P. chinensis* f. *alba*, consistent with the previous classification based on morphology. However, *P. cernua* f. *plumbea* did not cluster with *P. cernua* but with *P. dahurica*. Moreover, *P. cernua* was clustered with *P. tongkangensis*, a natural hybrid swarm population hybridized with *P. cernua* based on random amplified polymorphic DNA (RAPD) and SNPs of cpDNA [18], consistent with the results of K2p distance method. The attribution of *P. cernua* f. *plumbea* and its relationship with *P. cernua* and *P. dahurica* remain to be further confirmed and studied. Clades with the blue branch indicate four Chinese distributed species (*P. chinensis, P. cernua, P. dahurica*, and *P. turczaninovii*), the roots of which are recorded in the provincial and higher standards of herbal medicine as a source of “Bai tou weng.” In the future, phylogenetic relationship analysis of genus *Pulsatilla*, combined with the results of chemical composition analysis, will provide important reference information for the evaluation of the medicinal resources of “Bai tou weng” in China.

The new species *P. saxatilis* clustered with *P. campanella* into a branch but was phylogenetically distant from the most morphologically similar species, *P. chinensis*. Considering the gross morphology, *P. saxatlis* was closely similar to *P. chinensis* in having 3-foliolate leaves and solitary, erect flowers but differed in having light blue, whitish-blue, or white (vs. violet) sepals and persistent 2–2.5 cm (vs. 3.5–6.5 cm) long styles. Moreover, the phylogenetically closest species *P. campanella* has 3-foliolate leaves, but lower leaflets have essentially the same shape as lateral segments of central leaflet, appearing as a pinnate; flower nodding before and at anthesis; and sepals blue-violet to lilac in color. The morphological differences of the genus *Pulsatilla* are primarily reflected in flower morphology and leaf characteristics. Species with nodding flowers and pinnately divided leaves are phylogenetically derived from those with erect flowers and palmately divided leaves [19].

*P. campanella* is distributed in western Xinjiang, *P. saxatlis* is now found in Liaoning Province, and *P. chinensis* is widely distributed in northeast China (including Liaoning), North China, Sichuan, etc. Four types of germination pores occur in pollen grains of the genus *Pulsatilla*, namely, tricolpate, pantocolpate, pantoporate, and ditype pollen (with tricolpate and dicolpate) [20]. Among them, *P. chinensis* is tricolpate and *P. campanella* and *P. saxatlis* are pantoporate. Moreover, the evolution trend of each type is as follows: tricolpate → pantocolpate → pantoporate. Therefore, to explore the relative species of *P. saxatlis*, further data support and analyses are required.

Both traditional morphological classification and DNA barcoding are designed to solve the problem of species classification and identification, so as to protect and sustainably utilize biodiversity resources more effectively. In this study, 10 high-variable region sequences were screened for the construction of phylogenetic trees, and none were completely consistent with the cp. gene phylogenetic tree. This is mainly reflected in the difference of phylogenetic relationship between *P. campanella* and *P. saxatilis*. The tree with higher consistency with cp. gene tree structure is based on *ccs*A_*ndh*D (Fig. S2-A), *rps*16_*trn*K-UUU (Fig. 8-A), and *ccs*A (Fig. S2-C). However, *trn*Y-GUA_*trn*D-GUC with the same high Pi value has different branch structure. A common problem is that the outgroup (*Anemone tomentosa*) of these trees is not well distinguished, but does not have a significant impact on intra-genus identification. Of the 10 selected sequences, only *rbc*L effectively identified outgroups. The remaining selected sequences also have a certain discrimination rate. Generally speaking, the higher Pi value, usually the higher consistency with cp. gene tree and higher bootstrap value. However, *rps*12-intron (Fig. S2-F), the sequence with the highest Pi, failed to build phylogenetic tree because most branches had zero bootstrap. Through sequence alignment, it was found that the sequences were highly consistent, and only *P. dahurica* sequence was significantly different from other sequences. Therefore, *rps*12-intron is not suitable as a bar code for the identification of *Pulsatilla* genus, but it can be considered for the exclusive identification of *P. dahurica*.

When a single sequence is used for species identification, it may not be able to solve all the problems at the same time, such as inter-genus and intra-genus differences, so it is still necessary to conduct multi-sequence combination and experimental verification. At present, plant DNA barcoding is developing from a single or a few DNA fragments to a combination of multiple DNA fragments, cp. genome data, etc., and has been widely used in many studies [21]. In particular, the great improvement of genome sequencing technology makes it possible for researchers to explore “genome super DNA barcoding”. For closely related species, complete cp. gene can provide more comprehensive gene difference information for effective identification. In this study, cp. genes of *Pulsatilla* were analyzed and the effective DNA sequences were selected. It is of great significance for the species classification and identification of *Pulsatilla*.

## Conclusions

We reported the cpDNA characteristics of a newly discovered endangered species, *P. saxatilis*, and compared the cpDNA of 10 reported plants in the same genus. The cpDNA of the *Pulsatilla* exhibit highly similar size, GC content, gene sequence, and function. Through a series of comparisons, it was determined that the closest species to *P. saxatilis* is *P. campanella*, which is the same as the conclusion based on pollen grain characteristics, but different from the *P. chinensis* determined based on morphological characteristics. The above results are of great significance for the identification and taxonomy research of *Pulsatilla*.

## Methods

### Plant material and DNA extraction

Fresh young leaves of *P. saxatilis* were collected from Baiyun Mountain in Fengcheng, Liaoning, China (E 81°89′30″, N 43°26′98″) and subsequently dried using silica gel. The voucher specimens were identified by professor Tingguo Kang (Liaoning University of Traditional Chinese Medicine, Dalian, China) and deposited at the herbarium of Liaoning University of Traditional Chinese Medicine (Reference number: 10,162,210,527,011). Total genomic DNA was extracted using a Plant Tissue Chloroplast DNA Extraction Kit (Genmed Scientific Inc., Arlington, MA).

### Sequencing, assembly, and gene annotation of cpDNA

The total genomic DNA was constructed in a sequencing library with a 350-bp insert using the NexteraXT DNA library preparation kit (Illumina, San Diego, CA). Further, double-terminal sequencing was performed on the library using the Illumina Novaseq 6000 sequencing platform. The raw data were edited using NGS QC Tool Kit v2.3.3 [22]. Furthermore, high-quality reads were assembled into cp. genome using a *de novo* assembler SPAdes v3.11.0 [23]. Subsequently, the genome was annotated using the PGA program [24], with *Pulsatilla dahurica* (NCBI accession number: MK860685.1) cp. genome as the reference. Finally, the edited GenBank annotation file was submitted to OGDRAW to draw an annotation map [25].

### Comparative genomic analysis of the cp. Genome

In order to investigate the sequence divergence of the cp. genome of *Pulsatilla*, the cp. genome sequences of the 10 *Pulsatilla* species were visualized using mVISTA (http://genome.lbl.gov/vista/mvista/submit.shtml), with *P. chinensis* as the reference; moreover, the cp. genome was compared using default parameters in Shufe-LAGAN mode. Furthermore, to clarify the level of sequence variation, MEGA 6.0 software [26] was used to calculate SNP variation and K2p distance in *Pulsatilla* cp. genome.

To explore the diverging hotspot regions in *Pulsatilla* species and facilitate their utilization in identification, coding sequences (CDS), introns, and IGS of the cp. genome were extracted, and the sequences were compared using MAFFT v7; furthermore, the nucleotide and haplotype diversity were analyzed using DNAsp v6 software [27]. Phylogenetic trees were constructed for several sequences with higher Pi values. MAFFT v7 was used for cp. genome comparison, and IQ-TREE (v1.6.12) was used for optimal nucleotide model screening and ML tree analysis.

### Analyses of codon preferences

Codon usage bias analysis and calculation of the RSCU values were performed in the program CodonW v1.3. An RSCU value > 1 indicates frequent codon bias usage and a preferred codon, an RSCU value < 1 indicates less codon bias usage, and an RSCU value of 1 indicates no codon usage preference.

### Analysis of evolutionary selection pressure

Firstly, the homologous gene sequences of 10 *Pulsatilla* species were compared with MAFFT v7.429 (the default parameter was selected). And a well-aligned fasta format sequence file was obtained. Then use the software IQTREE 1.6.12 to generate the tree file. Finally, the codeml program of PAML V4.9 was used to calculate the Ka, Ks, and Ka/Ks values, and the results were sorted out and visualized.

### Repeat sequence and SSR analysis

SSRs were identified using MISA software (https://webblast.ipk-gatersleben.de/misa/), and the minimum repeats of single nucleotide, dinucleotide, trinucleotide, tetranucleotide, pentanucleotide, and hexanucleotide sequences were set to 10, 5, 4, 3, 3, and 3, respectively [28].

Dispersed repeats (forward, reverse, palindrome, and complementary) were identified using the online software REPuter (https://bibiserv.cebitec.uni-bielefeld.de/reputer/), and the minimum repeat size was set to 30 bp and hamming distance to 3 [29].

Tandem repeats were identified by running a web-based tandem repeat finder (https://tandem.bu.edu/trf/trf.html), in which the similarity percentage of two repeat copies was at least 90% and the minimal repeat size was 10 bp. The alignment parameters were set to 2, 7, and 7 for matches, mismatches, and indels, respectively.

### Phylogenetic analysis

cp. genome alignment was performed using MAFFT v7 [30], and the alignment gaps were stripped using Gblocks contrast. A total of 25 cp. genomes were aligned. Phylogenetic trees were inferred using ML and Bayesian inference (BI) methods. IQ-TREE (v1.6.12) was used for ML tree analyses. The phylogenetic analyses used the best-fitting models of nucleotide substitution selected in ModelFinder [31] under the Bayesian information criterion (BIC), and the optimal nucleic acid replacement model used was TVM + F + R2. Furthermore, BI was performed using MrBayes v3.2.6 [32]. The Markov chain Monte Carlo (MCMC) analysis was run twice for 5,000,000 generations. Every 1,000 generations were counted. The first 25% of the trees corresponding to the “burn-in” period were discarded, and the remaining three parts were used to construct the majority-rule consensus tree. According to BIC, the optimal nucleic acid replacement model used was GTR + F + I + G4.

### Divergence time analysis

cp. genome alignment was performed using MAFFT v7, and then blurred areas are modified using Gblocks contrast (removing locations containing comparison gaps). The modified sequences are used to construct phylogenetic trees. We selected four nodes to determine the divergence time: (1) *Panax ginseng* and *Potentilla chinensis* diverged 118 Mya (range, 111.4–123.9 Mya); (2) *Panax ginseng* and *Pulsatilla campanella* diverged 129 Mya (range, 126.0–132.4 Mya); (3) *Pulsatilla campanella* and *Anemoclema glaucifolium* diverged 26.0 Mya (range, 9.4–40.8 Mya) ; (4) *Pulsatilla campanella* and *Hepatica falconeri* diverged 28.2 Mya (range, 9.7–34.4 Mya) [33].

With reference to the above nodes, we used BEAST v2.6.2 to make divergence time inferences for cp. genome sequences of 25 species. Use Strict Clock as the inference method; In the MCMC operation, 10,000,000 generation tests are performed. In the process of merging tree, statistics are made every 1000 generations. At last, the first 25% trees are discarded as aging trees; The rest of the trees are merged to infer the divergent times represented by the tree structure and nodes. Use Tracer 1.7.1 to view the tree file with the ESS parameter > 200. Finally, use FigTree v1.4.4 to view the tree results and beautify them [34]. 

### Electronic supplementary material

Below is the link to the electronic supplementary material.

Supplementary Material 1

Supplementary Material 2

Supplementary Material 3

Supplementary Material 4

Supplementary Material 5

## Data Availability

The datasets generated and/or analyzed during the current study are available in the NCBI repository, https://www.ncbi.nlm.nih.gov/. Accession numbers: OP729488.

## Abbreviations

BI: Bayesian inference
BIC: Bayesian information criterion
CDS: Coding sequences
cpDNA: chloroplast DNA
IGS: Intergenic spacers
IRs: Inverted repeats
Ka: Nonsynonymous substitution rate
Ks: Synonymous substitution rate
LSC: Large single-copy
ML: Maximum likelihood
mtDNA: mitochondrial DNA
PCGs: Protein-coding genes
RSCU: Relative synonymous codon usage
SSC: Small single-copy
SSRs: Simple sequence repeats
