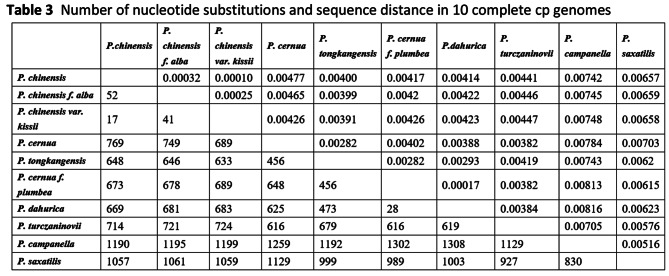# Correction: Comparative analysis of chloroplast genomes of *Pulsatilla* species reveals evolutionary and taxonomic status of newly discovered endangered species *Pulsatilla saxatilis*

**DOI:** 10.1186/s12870-024-05315-x

**Published:** 2024-06-29

**Authors:** Hefei Xue, Yanping Xing, Che Bian, Wenjuan Hou, Wenxiao Men, Han Zheng, Yanyun Yang, Xixiang Ying, Tingguo Kang, Liang Xu

**Affiliations:** 1 School of Pharmacy, Liaoning University of Traditional Chinese Medicine, Dalian, 116600 China; 2 Key Laboratory of Traditional Chinese Medicine Research and Development of Hebei Province, Institute of Traditional Chinese Medicine, Chengde Medical University, Chengde, 067000 China; 3 National Resource Center for Chinese Materia Medica, China Academy of Chinese Medical Sciences, Beijing, 100700 China; 4 State Key Laboratory of Dao-di Herbs, Beijing, 100700 China

**Correction: BMC Plant Biol 24, 293 (2024)**

10.1186/s12870-024-04940-w

Following publication of the original article [1], the authors identified errors, specifically:

- Figure 7 – Incomplete image that seriously affects the completeness and accuracy of the figure.
- Figure 8 – Extra horizontal line was spotted.
- Table 3 – Confusing format due to inappropriate column width.

The corrections do not affect the overall Conclusion of the article but need to be corrected for the completeness and accuracy of the content of the article.

The original article [1] has been corrected.

Incorrect Figure 7

**Fig. 7:**
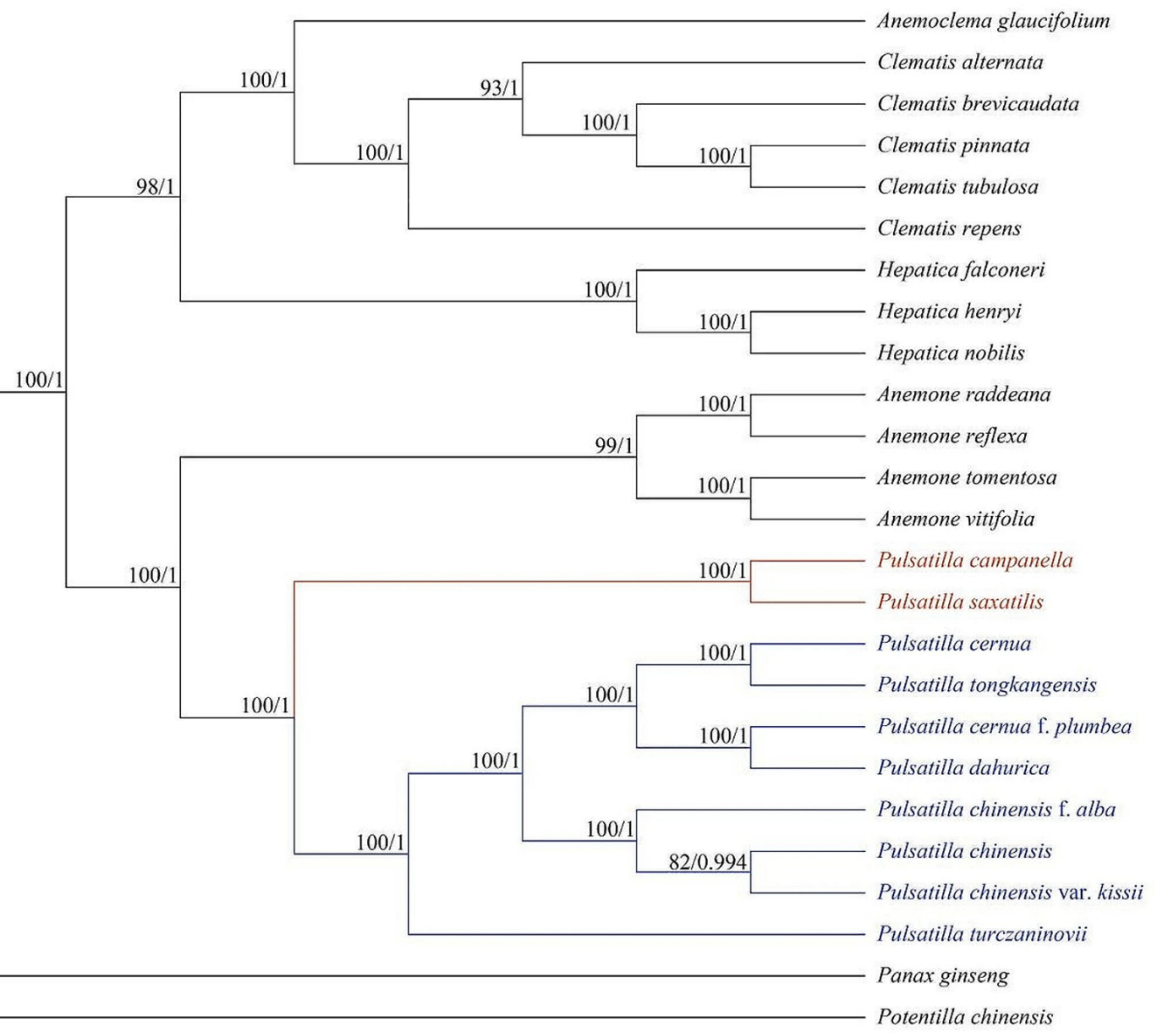
Phylogenetic tree of *Pulsatilla saxatilis* and 24 other species using maximum likelihood (ML) and Bayesian inference methods based on the complete cp. genome sequences. Number of the branches indicates ML bootstrap support value/Bayesian posterior probability

Correct Figure 7

**Fig. 7:**
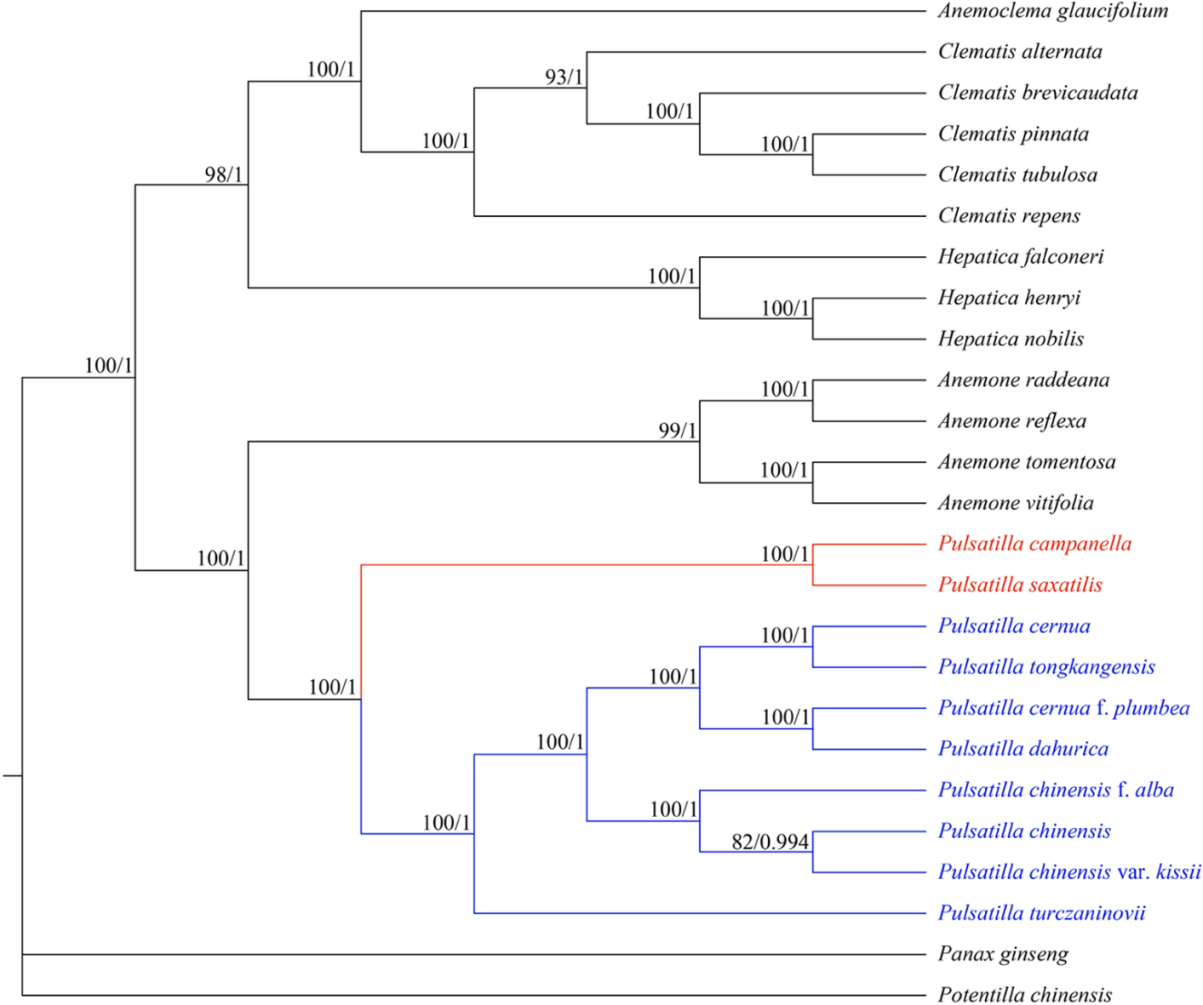
Phylogenetic tree of *Pulsatilla saxatilis* and 24 other species using maximum likelihood (ML) and Bayesian inference methods based on the complete cp. genome sequences. Number of the branches indicates ML bootstrap support value/Bayesian posterior probability

Incorrect Figure 8

**Fig. 8:**
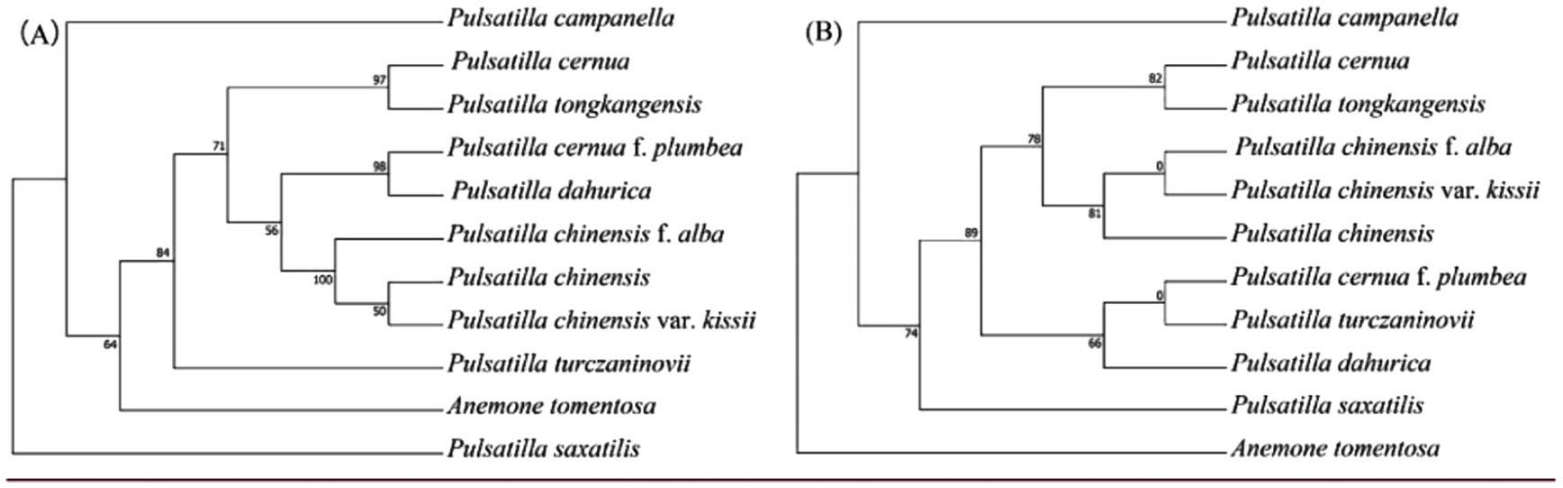
Phylogenetic trees of 10 *Pulsatilla* species and outgroup Anemone tomentosa using maximum likelihood (ML) based on *rps*16_*trnK*-UUU (**A**) and *rbcL* (**B**). Number of the branches indicates ML bootstrap support value

Correct Figure 8

**Fig. 8:**
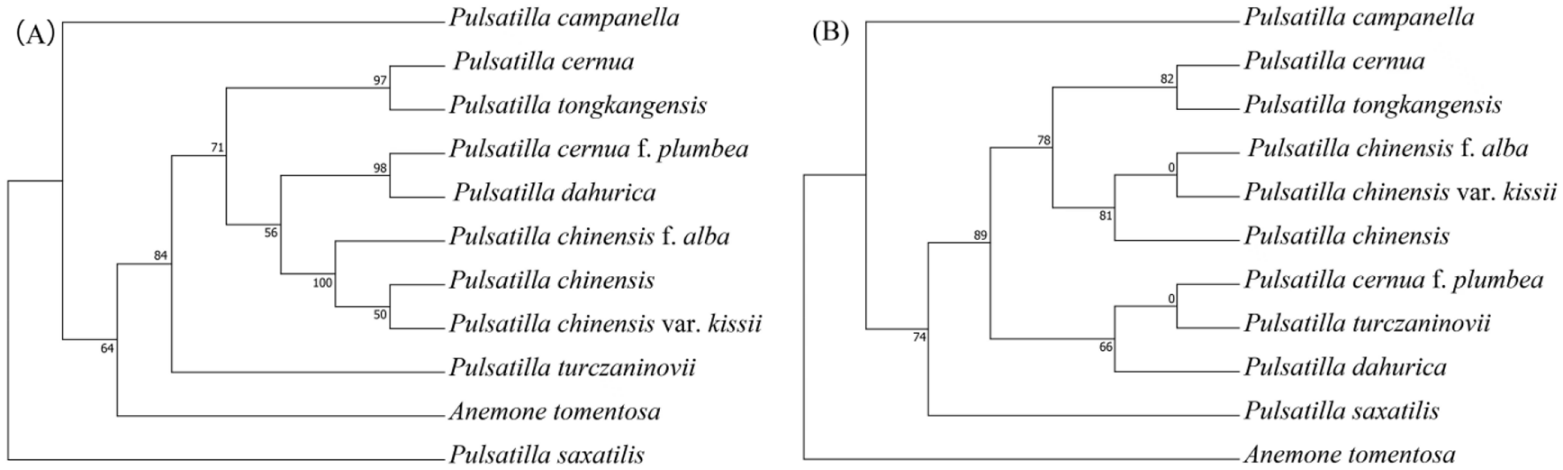
Phylogenetic trees of 10 *Pulsatilla* species and outgroup Anemone tomentosa using maximum likelihood (ML) based on *rps*16_*trnK*-UUU (**A**) and *rbcL* (**B**). Number of the branches indicates ML bootstrap support value

With confusing format Table 3

**Figure:**
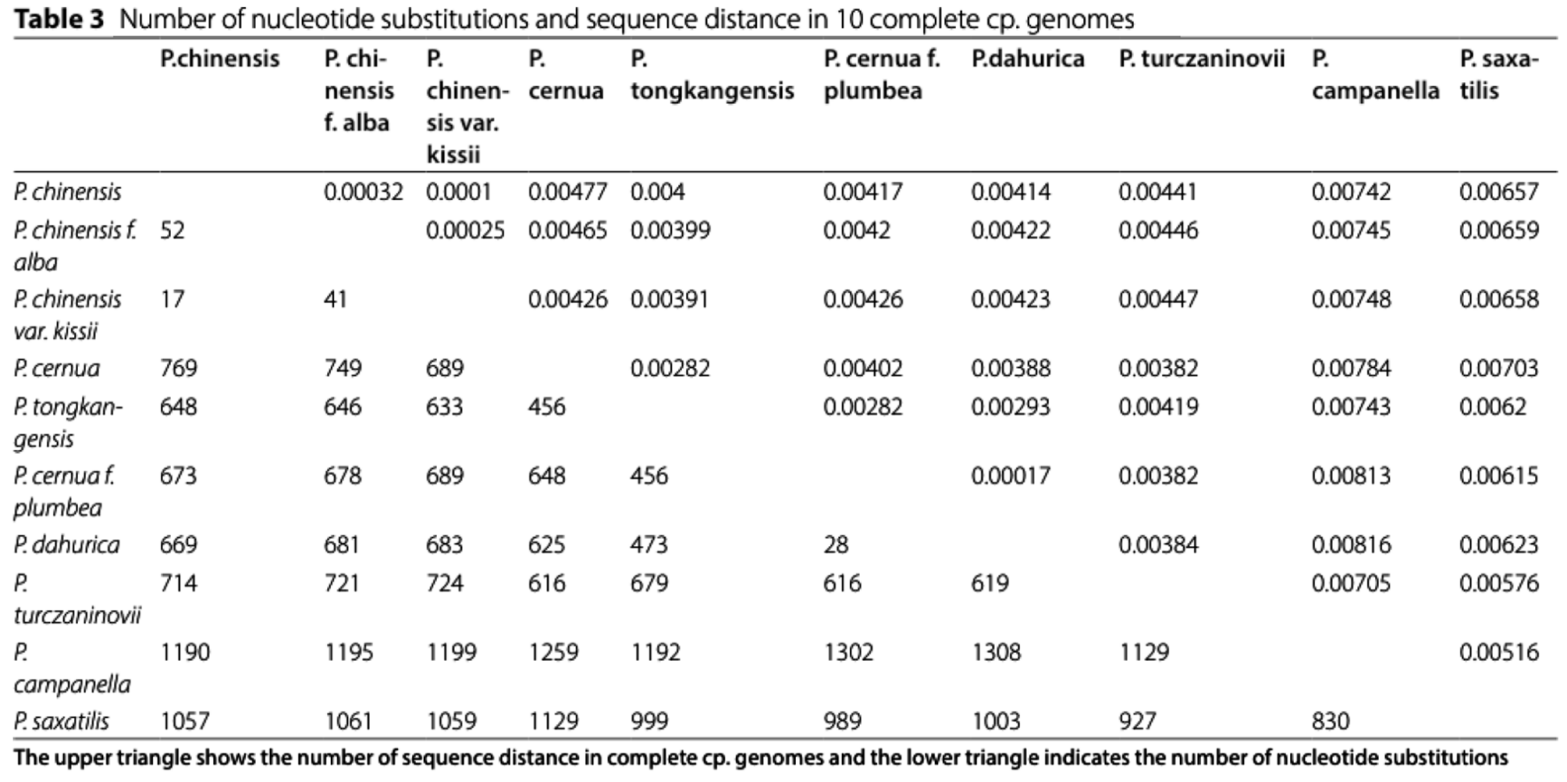

Clear format Table 3

**Figure:**