# Supplementary figures and images for: Comparative analysis of chloroplast genomes of Pulsatilla species reveals evolutionary and taxonomic status of newly discovered endangered species Pulsatilla saxatilis
Source: BMC Plant Biol. 2024 Apr 17;24:293. doi: 10.1186/s12870-024-04940-w (PMC11022354; doi:10.1186/s12870-024-04940-w)

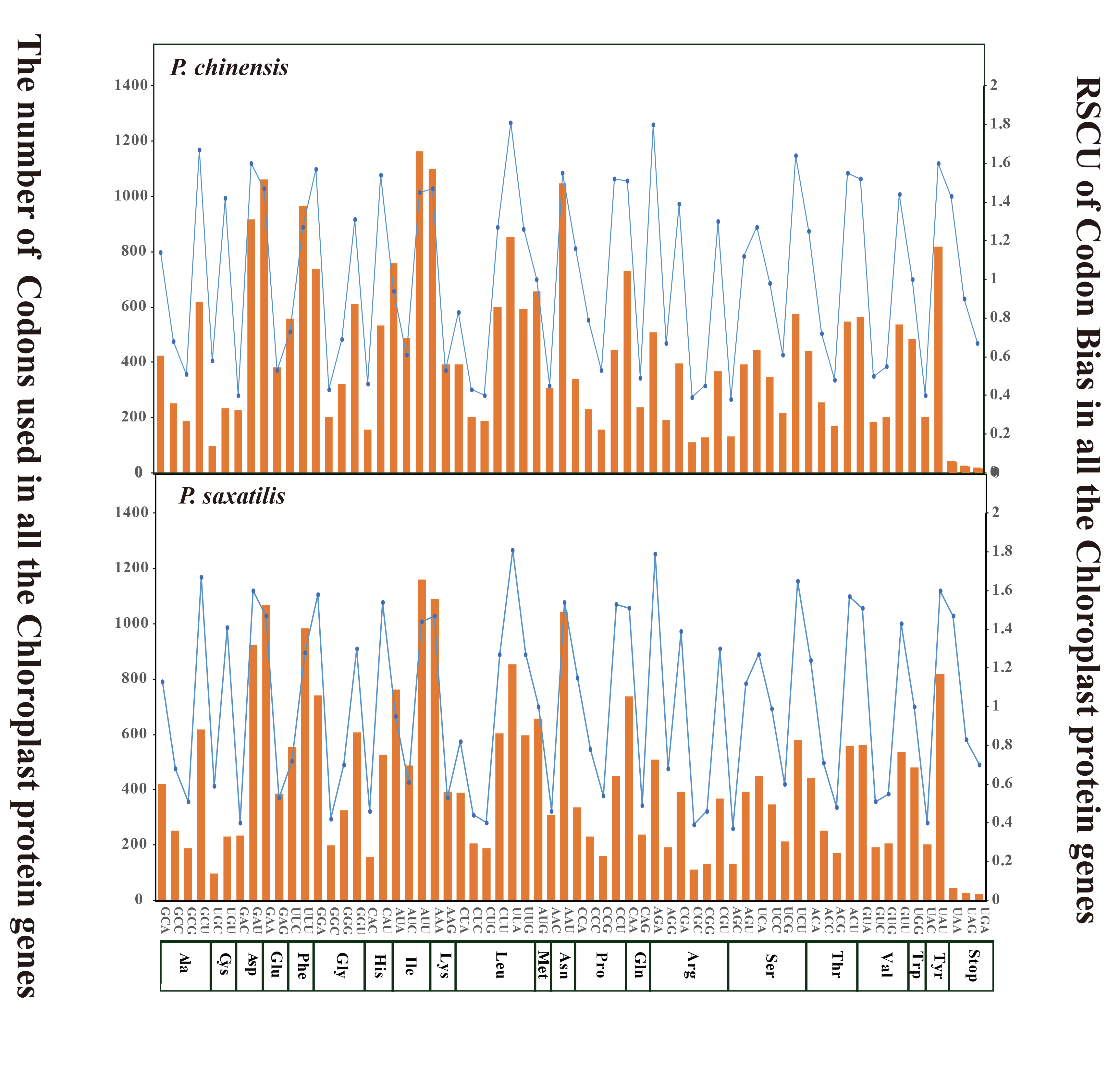

Supplement: Supplementary file 1 — Supplementary Material 1 [file 12870_2024_4940_MOESM1_ESM.jpg]

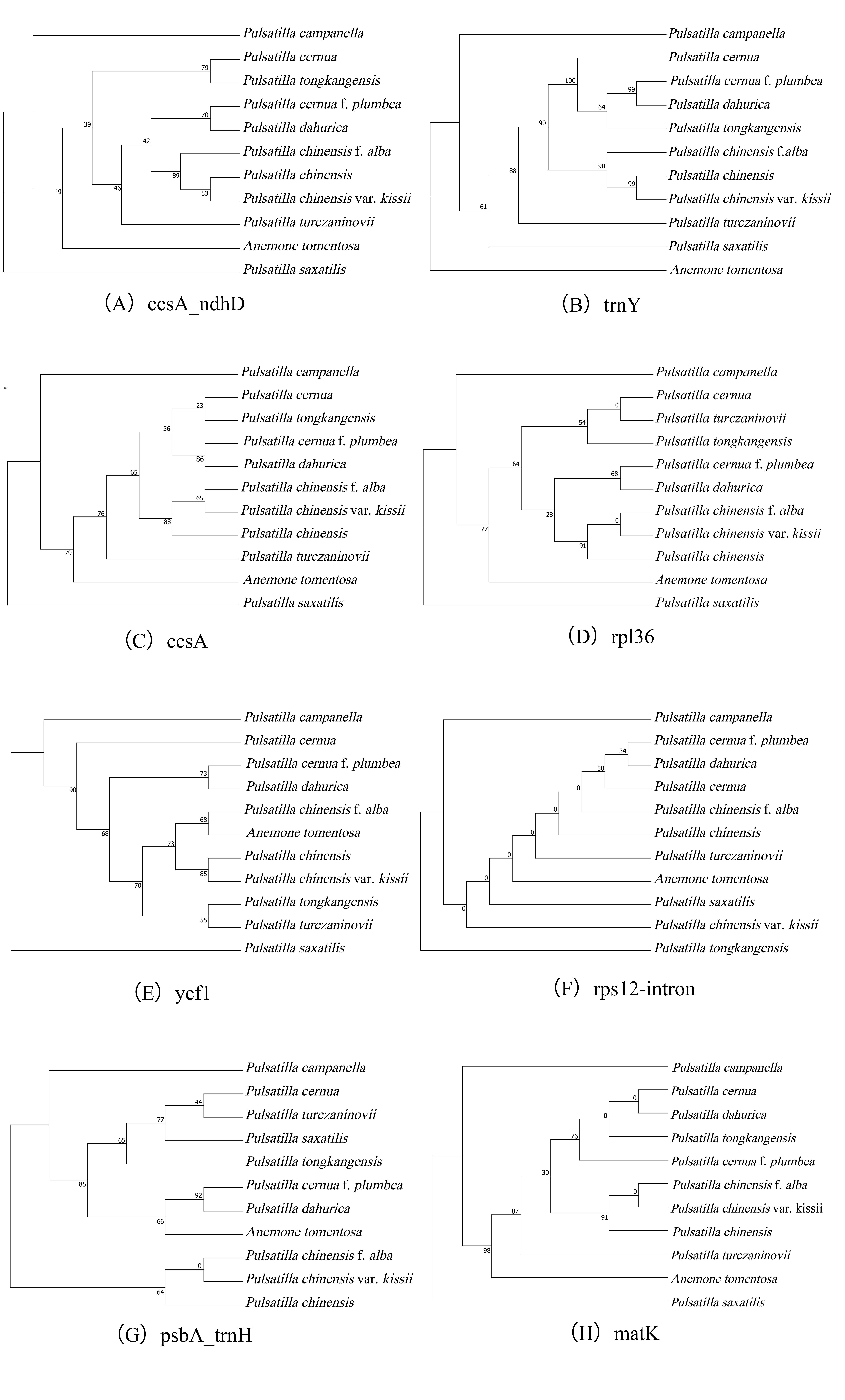

Supplement: Supplementary file 2 — Supplementary Material 2 [file 12870_2024_4940_MOESM2_ESM.jpg]
