# Supplementary material for: Comparative analysis of chloroplast genomes of Pulsatilla species reveals evolutionary and taxonomic status of newly discovered endangered species Pulsatilla saxatilis
Source: BMC Plant Biol. 2024 Apr 17;24:293. doi: 10.1186/s12870-024-04940-w (PMC11022354; doi:10.1186/s12870-024-04940-w)
Supplement: Supplementary file 3 — Supplementary Material 3 [file 12870_2024_4940_MOESM3_ESM.docx]

Fig. S1 Codon usage bias of *Pulsatilla saxatilis* and *P chinensis*

Fig. S2 Phylogenetic tree of 10 *Pulsatilla* species and outgroup Anemone tomentosa using maximum likelihood (ML)

Table S1 Common gene Haplotype Nucleotide Diversity

Table S2 KaKs ratio of 10 *Pulsatilla*
